# Integrating youth mental health practice nurses into general practice: effects on outpatient mental health care utilization among children and adolescents

**DOI:** 10.1007/s00787-024-02619-z

**Published:** 2024-12-12

**Authors:** Lukas B. M. Koet, Sanne Verhoog, Özcan Erdem, Heike Gerger, Patrick J. E. Bindels, Evelien I. T. de Schepper, Wilma Jansen

**Affiliations:** 1 Department of General Practice, Erasmus MC, University Medical Centre Rotterdam, Doctor Molewaterplein 50, Rotterdam, 3000 CA the Netherlands; 2 Department of Clinical Psychology, Open University, Heerlen, the Netherlands; 3 Department of Public Health, Erasmus MC, University Medical Centre Rotterdam, Rotterdam, the Netherlands; 4 Department of Research and Business Intelligence, Municipality of Rotterdam, Rotterdam, the Netherlands; 5 Department of Social Development, City of Rotterdam, Rotterdam, the Netherlands

**Keywords:** (MESH): Adolescent, Child, General practice, Nurse specialists, Mental disorders, Cost analysis

## Abstract

Integration of child mental health services in general practice may improve early detection and treatment and reduce strain on specialized services. In this study we investigated whether outpatient mental health care utilization and associated costs in children and adolescents were affected by the introduction of youth mental health practice nurses (YMHPNs) in general practice. We linked healthcare data of the Rijnmond Primary Care Database to municipal registry data on child outpatient mental health care expenditures between 2019 and 2022. Using mixed models, we assessed if the presence of a YMHPN in practices was associated with outpatient mental health care utilization. Our cohort consisted of 33,971 children aged 0–17 years registered in 38 general practices in Rotterdam, the Netherlands. 5.5% of these children attended outpatient mental health services between 2019 and 2022. The proportion of children utilizing outpatient mental health care and associated costs increased over time. After correction for practice demographics and trends over time, the presence of a YMHPN in a practice was associated with small non-significant reductions in the number of children receiving outpatient care (Rate Ratio = 0.99, 95%CI 0.92 to 1.06) and associated costs (-395.80 euros 95%CI -1431.27 to 639.67) compared with practices without YMHPN. Considering the study limitations, we cautiously concluded that the introduction of YMHPNs in general practice was not associated with significant changes in outpatient mental health care utilization one to four years after implementation. Future studies should elucidate the long-term impact and underlying changes in pathways to care due to the introduction of the YMHPN.

**Supplementary Information:**

The online version contains supplementary material available at 10.1007/s00787-024-02619-z.

## Introduction

Worldwide there has been an increasing demand for child mental health services in the last decades [1, 2]. However, mental health services often lack resources to handle this increasing demand resulting in long waiting-lists and rejection of referrals [3]. The limited access to mental health services can be especially problematic in cases where early detection and treatment of the problem could have prevented problems later in life [4]. In addition, there is evidence that referral of children with limited or no mental health impairment to child mental health care is at least partially responsible for the observed strain on these services [2]. To address the problems associated with restricted access to mental health care services and to relieve overburdened specialized services, it has been suggested to integrate mental health services into primary care [5]. Available evidence suggests that integrated care models may offer several benefits compared to usual care, including improved accessibility and better mental health outcomes [6]. 

In the Netherlands, all health care for children below 18 years is reimbursed. Primary care for children and adolescents is delivered by general practitioners (GPs). All children and adolescents are registered at the practice of their local GP and typically only change practices when moving further away. Children and their caregivers can seek help for mental health problems from their GP, who can either provide basic treatment themselves or refer to specialized services if needed. However, many GPs feel they lack confidence and sufficient time to manage child mental health problems and often choose to refer rather than to manage these problems themselves [7–9]. In the Netherlands children and adolescents referred to mental health services are typically treated on an outpatient basis, mostly provided by large mental health institutions. GPs experience difficulties in the referral process (e.g., selecting the appropriate provider) and regularly see their referrals rejected [7]. In the Netherlands, there is no direct access to child mental health services. As such, children need a referral to access mental care, which can be provided by different health professionals including GPs, local support teams, and hospital pediatricians. In Rotterdam, GPs are responsible for most mental health care referrals.

Since 2015 a specialized position of ‘youth mental health practice nurse’ (YMHPN)^1^ has been introduced into general practice [10]. The GP can refer children to the YMHPN when they present with psychosocial problems. The aims of introducing the YMHPN are to improve early treatment of child mental health, improve quality of referrals, and to reduce unnecessary referrals to specialized mental health care by offering basic care [10]. YMHPNs are professionals with experience in youth care and can have diverse backgrounds including psychiatric nursing, psychology, and social work. YMHPNs work within general practices and provide a variety of tasks, such as problem clarification, support for parenting, psycho-education, short-term basic treatment of mental health problems, and specific family interventions [10]. Furthermore, they assist GPs by providing information and advice, serve as liaisons with external parties such as schools, social support teams, and specialized mental health services, and refer children on behalf of the GP when specialized care is needed [11]. Usually, there is no waiting list for the YMHPN [7, 12]. Unlike GPs, YMHPNs are not licensed to prescribe medication. Although the number of initiatives to integrate mental health services into primary care is increasing worldwide [6], the position of the YMHPN in the Netherlands is not directly comparable to other initiatives.

Also, in the Netherlands the number of children receiving mental care services has increased substantially in the last two decades, as well as the needed budgets [13]. It is unclear what effects the introduction on the YMHPNs has had on child mental health care utilization. On the one hand the introduction of YMHPNs may prevent unnecessary referrals and lead to more early treatment, which may prevent worsening of mental problems and reduce the need for more specialized care. On the other hand, the introduction of YMHPNs may reduce barriers to mental care, effectually leading to more children receiving mental health care, either in general practice or in a specialized setting. It is also possible for these effects to co-occur. To date, it remains unclear whether the introduction of YMHPNs in general practice leads to different outpatient mental care utilization among affected children.

In this study, we first investigated whether outpatient mental health care utilization changed over time. Secondly, we evaluated which child characteristics are associated with outpatient mental health care utilization. Thirdly, we investigated whether the introduction of YMHPNs into general practice is associated with changes in outpatient mental healthcare utilization and costs for outpatient mental healthcare.

## Methods

### Study design and population

We performed a retrospective population-based cohort study using GP medical records and municipal registry data of children and adolescents aged 0–17 years in Rotterdam between January 1st, 2019 and December 31st, 2022.

## Setting

Rotterdam is the second-largest city of the Netherlands, with a population of 670,000. It has a large community of ethnic minorities and the highest percentage of children living in a low-income household of the Netherlands [14]. In 2019, after performing a pilot-project, the municipality of Rotterdam implemented the integration of child mental health services into primary care by introducing youth mental health practice nurses (YMHPNs) into several general practices (YMHPN-project) [15]. In the practices participating in the project, YMHPNs work typically 1 to 2 days per week. All starting YMHPNs in Rotterdam are obliged to follow a 1-year post-graduate training and receive continued education. Salary costs of the YMHPNs are paid by the municipality. The number of general practices participating in the YMHPN-project has steadily increased. In 2019 approximately 20% of children in the municipality Rotterdam were registered in a practice with a YMHPN which increased to approximately 50% in 2022.

### Data sources and data linkage

We linked data of our GP database (Rijnmond Primary Care Database) with municipal registry data on youth care expenses. Practices participating in the YMHPN-project also joined the Rijnmond Primary Care Database (RPCD) for evaluation purposes. The RPCD is a region-specific derivative of the Integrated Primary Care Information (IPCI) database, covering the Rotterdam metropolitan area [16]. It contains pseudonymized routinely collected medical data of general practice patients. The RPCD database has extensive quality control measures [16]. New practices joining the RPCD start providing data one year after joining the database (run-in period). The municipal registry data contains information on all youth care expenditures (e.g., outpatient and inpatient mental health services, foster care) of all children aged 0–17 living in Rotterdam from 2019 onwards. From age 18 onwards health care costs are reimbursed by health insurance, and therefore no information for adults was available.

We selected data on outpatient mental health care only because YMHPN typically refer their patients to outpatient care and the care delivered by YMHPN may theoretically substitute outpatient care delivered for non-complex mental problems [11, 17]. Outpatient mental health care was defined by any outpatient psychosocial care, one-on-one or in groups, delivered by any mental health institution or practice. The RPCD and municipal registry do not contain robust information on expenditures in general practice and medication costs because these are covered by health insurances. General practice expenditures and medication costs related to child mental health problems are relatively low compared to costs for more complex care such as outpatient mental health care. For these reasons, we did not take these costs into consideration in our analyses. The municipality provided information regarding the specific general practices in which a YMHPN was employed, along with the corresponding start dates for each practice. General practices in Rotterdam participating in the RPCD were informed about the study goals and were asked to give consent for data linkage. Out of 47 participating practices, we received consent from 38 practices. Data linkage was performed by a trusted third party. The authors were not involved in the linkage process.

### Data extraction

For every child we extracted from the municipal registry monthly data on whether a child received outpatient mental health care, and the associated monthly costs in euros. These costs are estimates of the expenditures based on the indicated care at start of the treatment. Cost estimates are based on price agreements between the municipality and the care providers. If the treatment plan changed during the treatment trajectory (e.g., prolonged or more intense treatment was needed), new estimates for the subsequent months were available. The estimated costs are assumed to be a good representation of actual costs. Additionally, we extracted from the RPCD monthly information on the child’s age, sex, and social deprivation status. Social deprivation status is a summary score (deprived or non-deprived) calculated for every postal code using deprivation indicators (i.e., benefit recipients, residents with low income, surrounding address density and non-western foreigner) provided by the Dutch Healthcare Authority (NZA) [18]. 

### Analysis

All analysis were performed in R (version 4.3.1; glmmtmb package) [19]. We compared differences in outpatient healthcare utilization between boys and girls, and children living in deprived and non-deprived areas using student t-test and chi-square test. To assess the association between the presence of YMHPNs and outpatient mental health care utilization we used aggregated data per month per practice. We used aggregated data because we regard the introduction of the YMHPN as a practice level intervention. The effects of a YMHPN in a practice may be direct (e.g., consultations with the YMHPN take place) but also indirect (e.g., increased awareness in the practices of mental health problems, GP consults the YMHPN for advice on treatment plan with no appointments between child and YMHPN taking place). We used mixed-effects models estimating the association between the presence of YMHPNs and the two outcomes of interest (1) the number of children receiving outpatient mental health care, and (2) the costs for outpatient mental health care per practice, while accounting for the correlation between monthly measurements of each practice. We used Poisson mixed-effects models for the number of children receiving care and linear mixed-effects models for the total costs for outpatient mental health care per practice. In the fixed-effects part we allowed for a linear effect of time (In months ranging from 0 in January 2019 to 47 in December 2022). We corrected for practice characteristics (i.e., number of children per practice, average age of children within practices, proportion of boys, and proportion of children living in deprived areas). In the Netherlands the number of referrals to child mental care decreased significantly in 2020 due to the COVID-19 pandemic [20]. Therefore, we included a dummy variable (March-December 2020) to adjust for possible confounding effects of the first year of the pandemic. In the random-effects structure we included random intercepts and random linear time per practice. Residual plots were used to validate the models’ assumptions.

### Sensitivity analyses

Since participation in the YMHPN-project was not random (i.e., practices could decide themselves whether to participate or not) participating practices may differ in terms of care utilization from non-participating practices regardless of the presence of a YMHPN. To assess possible confounding effects of unmeasured differences between participating and non-participating practices, we repeated the analyses using only practices that participated in the YMHPN-project. To assess possible confounding effects of a dynamic cohort (e.g., practices joining the cohort during the study period) we repeated the above analysis using only practices that provided at least three years of follow-up information.

### Ethics and data availability

Under Dutch GDPR law, this study does not require ethical approval. The RPCD is a pseudonymised, opt-out database, stored confidentially on a local server of ErasmusMC. Patients of participating practices are informed by their GP about the participation of the practice, and that their information can be removed from the database on request. The Governance Board of the RPCD approved our study protocol (project-number 2020.013). We followed the RECORD guidelines for studies using routinely-collected health data [21]. Due to legal constraints, the data is not publicly available.

## Results

### Cohort characteristics

**Fig. 1:**
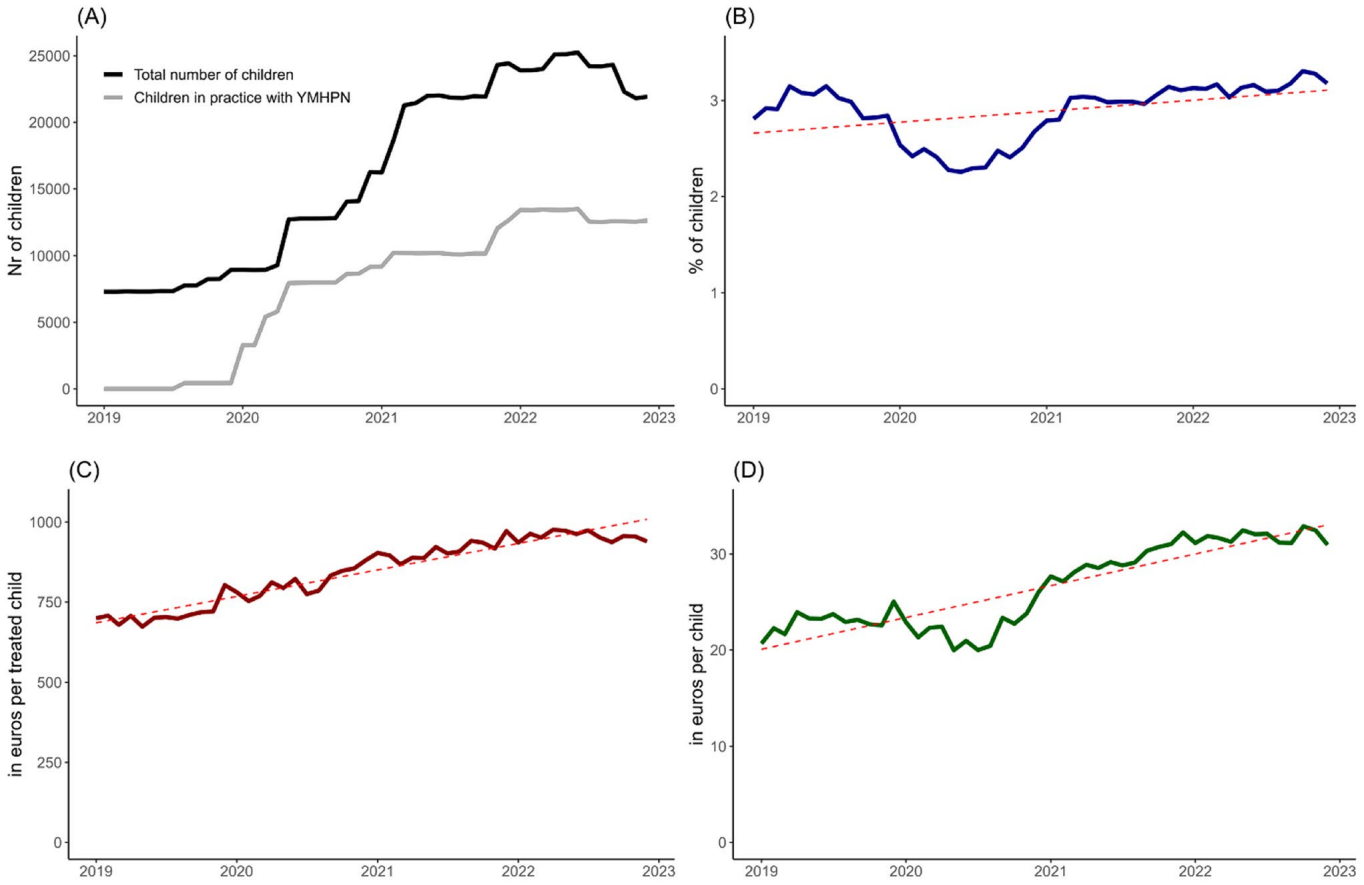
Changes in cohort size, mental health care utilization and costs over time *These graphs show monthly data on number of children in cohort (**A**), and monthly outpatient mental health care utilization (**B**), and associated costs (**C, D**). The dotted-line in figure b, c and d shows the simple regression line over time. The proportion of children in outpatient care was lower than expected in the first year of the COVID-19 pandemic. Consequently, average costs per child were also lower in the first year of the pandemic

38 general practices with a total of 33,971 children contributed to our cohort. At the start of the study in January 2019, the cohort consisted of 9 practices with 7718 children. Over time the number of practices providing information to the RPCD database increased, which can partly be explained by practices joining the YMHPN-project during the study period. (see Table 1; Fig. 1). Children were on average 8.6 years old (SD 5.1) and 49.3% were girls. In January 2019 a total of 28.4% of children lived in a deprived area which increased to 43.2% by December 2022. Changes in cohort demographics are also explained by more practices providing data to the RPCD over time (see Table 1; Fig. 1).

**Table 1:** Cohort demographics over time

	Total cohort size^a^	Number of children in practice with YMHPN^b^	Mean age (SD)	% Male	% living in deprived area
2019-01-01	7298	0	8.6 (5.2)	50.3	28.4
2019-07-01	7335	0	8.6 (5.1)	50.2	28.6
2020-01-01	8943	3278	8.5 (5.1)	50.3	27.6
2020-07-01	12,767	7983	8.5 (5.1)	50.6	40.5
2021-01-01	16,232	9195	8.6 (5.1)	51.0	44.3
2021-07-01	21,859	10,111	8.6 (5.0)	50.8	40.8
2022-01-01	23,898	13,414	8.6 (5.1)	50.8	40.1
2022-07-01	24,216	12,536	8.6 (5.1)	50.5	42.5
2022-12-01	21,927	12,624	8.7 (5.0)	50.6	43.2

^a^The cohort size increased over time due to more practices joining the RPCD. One practice was closed during follow-up. 7 practices had not yet provided follow-up information for the last months of 2022 at time of data-linkage. ^b^Over time more practice that employed a YMHPN joined the RPCD and some already participating practices started employing a YMHPN. One practice stopped employing a YMHPN during follow-up

### Practice characteristics

The number of children per practice ranged from 205 to 2150 children. The percentage of children living in socially deprived areas varied by practice, ranging from 1.8 to 91.4%. There were large differences in the mean of monthly outpatient mental health care utilization between practices ranging from 0.9 to 5.6% of children. Taking into account the number of children per practice, the average monthly costs of practices ranged from 9.33 to 41.38 euro per registered child. Twenty practices never employed a YMHPN in their practice during the study period. Nine practices started employing a YMHPN during the study period. Eight practices had a YMHPN during the complete study period. One practice had a YMHPN at the start of the study period but stopped with the project during follow-up. Trends over time in these outcomes seemed to vary per practice (see eFigure 1). Although utilization rates and mean costs tended to increase over time, patterns over time varied between practices. No uniform changes were seen either on outpatient health care utilization or on associated costs after a YMHPN started in a practice (eFigure 1).

### Utilization and costs of outpatient mental health care

On average 5.5% of children attended outpatient mental health services during the study period (mean follow-up per child = 24.1 months). The monthly percentage of children attending these services increased from 2.8% in January 2019 to 3.2% by December 2022. However, during the year of the Covid-19 pandemic this rate dropped temporarily to a minimum of 2.3% in June 2020 (Fig. 1B). Monthly rates were higher for boys than for girls (average monthly rate 3.3% vs. 2.6%, *p* < 0.001). Monthly rates were lower for children living in a social deprived area than for those in non-deprived areas (average monthly rate 2.4% vs. 3.3%, *p* < 0.001).

**Fig. 2:**
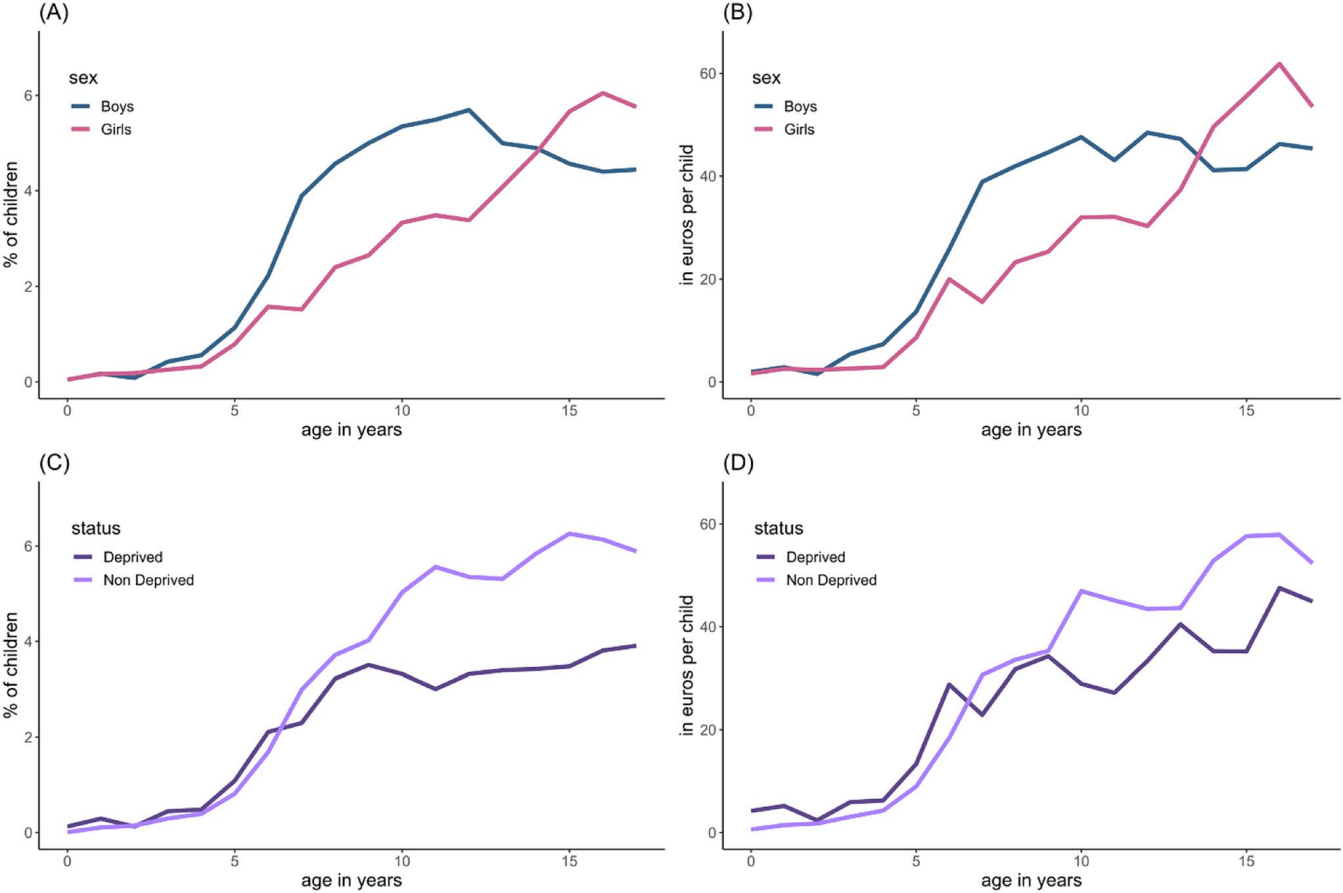
Outpatient mental health care utilization per sex, age and deprivation status

The average monthly cost for outpatient mental health care increased from 699.23 euros in January 2019 to 939.47 euros per treated child by December 2022 (Fig. 1C). Averaged monthly cost across all children, including those who attended outpatient care and those who did not, increased from 23.66 euro per child in January 2019 to 34.25 euro per child by December 2022 (Fig. 1D). However, during the first year of the COVID-19 pandemic average costs temporarily decreased to 22.55 euros per child in May 2020. The average monthly cost for boys was higher than for girls (30.62 vs. 25.70 euros, *p* < 0.001). In girls, the cost for outpatient mental health care peaked at ages 14 to 17. For boys, mean cost increased until age eight, after which these remained stable (see Fig. 2). The average monthly cost was lower for children living in a socially deprived area than for children from non-deprived areas (25.77 vs. 30.06, *p* < 0.001). Figure 2 and eFigure 2 show mental health care utilization and associated costs per sex, age and deprived area.

### Associations between the presence of YMHPNs and outpatient care utilization and associated costs

In the analysis assessing the association of the presence of a YMHPN in a practice and outpatient mental health care utilization, most practice demographics (i.e., number of children per practice, average age of children within practices, and proportion of children living in deprived areas) were relevant predictors of the number of children receiving outpatient care (Table 2). Trend over time was also a relevant predictor for care utilization, with utilization increasing over time. The first Covid-19 year (March-December 2020) was associated with a significant reduction in outpatient care utilization (Rate Ratio [RR] = 0.88, 95%CI 0.84–0.92). After adjustment for these confounders, the presence of a YMHPN was associated with a minimal non-significant reduction in number of children receiving outpatient care (RR = 0.99, 95%CI 0.92–1.06).

**Table 2:** Poisson mixed model results of monthly outpatient mental health care utilization per practice

Model 1. Modelled monthly rate of children receiving outpatient mental care per practice			
*Predictors*	*Rate Ratios*	*CI*	*p*
(Intercept)	0.022	0.02–0.03	< 0.001
Yearly trend over time^a^	1.06	1.01–1.11	0.024
% Living in social deprived area^b, c^	0.99	0.99–0.99	< 0.001
Mean age in years^c^	1.14	1.03–1.27	0.015
% Male^b, c^	1.00	0.98–1.03	0.855
YMHPN in practice	**0.99**	**0.92–1.06**	**0.695**
First Covid-19 year^d^	0.88	0.84–0.92	< 0.001

^a^Modelled linear trend every year increase over time is associated with increasing number of children receiving outpatient care. ^b^In percentage per practice, the shown estimate is the relative rate per percentage increase of the variable (e.g. 40 to 41% percentage of children living in deprived area) ^c^These variables are centered on their grand-mean average (e.g., average number of children registered per practice) to facilitate interpretation. ^d^ Dummy variable to correct for the period of March 2020 to December 2020 in which a strong decrease in mental health care utilization was observed

The modelled expected costs for an average practice of 678 children in January 2019 was 16,793 euros per month. In the analyses assessing the association of the presence of a YMHPN in a practice and costs for outpatient mental health care, most practice demographics (i.e., number of children per practice, average age of children within practices, and proportion of children living in deprived areas) and trend over time were relevant predictors for the total cost for outpatient care per practice (Table 3). The first Covid-19 year was associated with a reduction in monthly costs (-2333.39 euros 95%CI -2931.19 to -1735.60). After adjustment for these confounders, the presence of a YMHPN in a practice was associated with a small, non-significant reduction of costs for outpatient mental health care services of 395.80 euros (95%CI -1431.27 to 639.67) per month per practice.

**Table 3:** Linear mixed model results of monthly costs for outpatient mental health care per practice

Model 2. Modelled costs for total outpatient mental care per practice			
*Predictors*	*Estimates*	*CI*	*p*
(Intercept)	16793.10	12922.76–20663.43	< 0.001
Yearly trend over time^a^	1500.36	8.22–241.83	0.036
% Living in social deprived area^b, c^	-111.78	-165.91 – -57.64	< 0.001
Mean age in years^c^	3412.38	1755.19–5069.58	< 0.001
% Male^b, c^	-42.40	-354.51–269.72	0.790
YMHPN in practice	**-395.80**	**-1431.27–639.67**	**0.454**
Practice size (nr. of registered children)^c^	34.55	30.58–38.51	< 0.001
First Covid-19 year^d^	-2333.39	-2931.19 – -1735.60	< 0.001

^a^Modelled linear trend over time to allow for increasing costs for each year (e.g., inflation). ^b^In percentage per practice, the shown estimate is the increase in costs per percentage increase of the variable (e.g., 40 to 41% percentage of children living in deprived area) ^c^These variables are centered on the grand-mean average (e.g., average number of children registered per practice). ^d^Dummy variable to correct for the period of March 2020 to December 2020

### Sensitivity analyses

We performed sensitivity analyses in two subsets of practices. Although the estimates on the association found in the sensitivity analyses differ slightly from the main analysis including all practices (see eTable 1–2), the overall conclusions are in line with the main analysis. In the first sensitivity analysis including only practices participating in the YMHPN-project, the introduction of a YMHPN in a practice was associated with small non-significant reductions in the monthly number of children receiving care (RR = 0.94, 95CI% 0.87–1.02) and associated monthly costs (-575.86 euros, 95%CI -1787.18 to 635.46). In the second sensitivity analysis including only practices with at least 36 months follow-up, the presence of a YMHPN in a practice was also associated with small, non-significant reductions in the number of children receiving care (RR = 0.98, 95CI 0.90–1.07), and associated costs (-960.13 euros, -2205.74 to 285.48).

## Discussion

In our study we investigated whether the introduction of youth mental health practice nurses in general practice was associated with outpatient mental health services utilization and costs for outpatient healthcare in one to four years after implementation. Overall, outpatient health care utilization and associated costs increased steadily between 2019 and 2022. After adjustment for relevant confounders, including the COVID-19 pandemic, the presence of a YMHPN in a practice was associated with small, non-significant reductions of outpatient health care utilization and associated costs. Continued evaluation of the introduction of the YMHPN in general practice is needed to confirm the current findings and to assess whether longer-term effects differ from the shorter-term effects.

Worldwide utilization of mental health services has increased substantially among children and adolescents over the past decades [1, 22, 23]. Of the several types of care (e.g., inpatient, school-counselling), the increased utilization of outpatient mental health care is apparent [24]. In our study, we also saw a slow but steady increase in outpatient mental health care utilization in children and adolescents. Furthermore, we noticed a positive correlation between age and the percentage of children using outpatient mental health. Additionally, boys were more likely to receive care than girls. In contrast with our findings, previous research showed inconsistent associations between mental health service use and sex and age [25]. Although adolescent girls tend to experience more mental problems than adolescent boys [26], we noticed that before the age of 15 health utilization in boys is higher than in girls suggesting unmet needs of girls in this age category. In our study on average 5.5% of children attended outpatient mental health services during the study period. Although this seems relatively low considering the high prevalence of child mental health problems, it is comparable to mental care utilization in other high-income countries [27]. It is also in line with regional registry data which show that approximately 7% of children up to 17 years in Rotterdam receive one or more forms of youth care including among others outpatient mental care, help for dyslexia, and developmental problems [28]. 

Growing up in a socially deprived area has been shown to be an important risk factor for developing mental health problems and is associated with a lower well-being [29]. On the contrary, mental health care utilization is assumed to be lower among children and adolescents living in a socially deprived area than among children living in less deprived areas [30]. This is supported by our findings, where living in a deprived area was associated with less outpatient mental health service use and lower costs. This is worrisome and suggests that although children in socially deprived areas may suffer more from mental health problems, they are less likely to receive care for these issues. One of the goals of integrating mental health services into primary care is to improve accessibility of care for all children regardless of their background [31]. Future research should therefore investigate whether the availability of a YMHPN has differential effects on accessibility of mental health care for children with different socioeconomic, cultural and ethnic backgrounds.

In the past decades, several studies evaluated initiatives to integrate child mental health services into primary care. A meta-analysis of randomized clinical trials showed that integrated mental health care for children and adolescents was associated with improved health outcomes compared with usual care [6]. Additionally, integrated child mental care within primary care may have the potential to reduce disparities for vulnerable children (e.g., children from a minority or social-economically disadvantaged background) [31]. To our knowledge only a limited number of studies evaluated cost effects of such initiatives, using relatively small samples [32, 33]. As far as we are aware, this is the first large-scale study investigating the associations between integrated child mental health care into general practice and the utilization of outpatient mental health care and associated costs. Earlier studies investigating the effects of integrating mental health professionals into general practice in adult populations showed various results, with no clear evidence on whether this form of integrated care changed specialized care utilization and costs [34]. Overall, we found that integrating YMHPNs within general practices did neither lead to short term changes in the number of children receiving outpatient mental health care nor to significant changes in associated costs. It is important to note that we could only investigate overall associations. We were not able to investigate whether the presence of YMHPNs had different effects for specific subgroups (e.g., whether the presence of a YMHPN was associated with specific mental health problems being more or less often treated in outpatient care, or whether it was associated with less unnecessary referrals). Importantly, children registered in a practice with a YMHPN receiving outpatient mental care were not necessarily referred by the YMHPN. They could also be referred directly by their GP or other care providers. Importantly, large variation on both outcomes became apparent between practices which deserves further attention in future research.

YMHPNs can play an important role in lowering barriers to access services. Many barriers (e.g. limited access to mental services and fear of stigmatization) are shown to influence access to mental health services and gaps are still suspected between those who need care and those who receive care [27, 35]. In our study we did not have information on the number of children seen by YMHPNs, and as a result we do not know whether the introduction of the YMHPN led to more children being treated within general practice. However, the monthly rate of children receiving outpatient care did not change following the introduction of YMHPNs. Importantly, an earlier study showed that YMHPNs can successfully manage a substantial part of children without the need for additional referral to specialized care [17]. 

One of the presumed benefits of integrating child mental health services into primary care is that it improves early detection and treatment [6]. Integrated care may prevent mental health problems from worsening and reduce long-term negative outcomes and associated costs for the affected individual and society [4]. Additionally, integrated care (e.g., YMHPNs) may reduce referrals for non-complex problems and prevent unnecessary medicalization. To determine whether YMHPNs can indeed prevent long-term negative outcomes, studies with longer follow-up periods are necessary. The current study focused on the effects within the first four years of the introduction of the YMHPN. Longer-term effects may differ from the observed shorter-term effects. Therefore, further research is needed to investigate whether the introduction of the YMHPN led to long-term changes in mental health care utilization (e.g., both in absolute rate of mental health services use and on type of service use). Such research should preferably encompass all levels of Dutch mental health care, as well as costs for medication, which will allow stronger and more specific inferences. Besides the prevention of long-term negative outcomes, there might be other effects of introducing the YMHPN into general practice that are not captured in this study and might confound the overall found association, such as an increased interest in children’s mental health by the GP or a lower rejection rate of referrals to specialized mental health care. A study on the long-term effects could be extended with questionnaire data from GP practices, YMHPNs and children and their family members to investigate other effects from the introduction of the YMHPN. This data could also be used to investigate differences between practices and YMHPNs. Moreover, with questionnaire data it is possible to collect information on patient characteristics (e.g., clinical condition) and investigate the effect for different subgroups or certain interactions.

### Limitations

Our study has several limitations. First, it was not possible to investigate on the individual child level whether the introduction of the YMHPN led to changes in the number of children receiving help, because we did not have information on which children were seen by YMHPNs. As such, we were only able to investigate the overall effect of the availability of a YMHPN in a practice on outpatient care utilization. Nevertheless, we believe that this study offers valuable insights for practice and policy as this is the first study to evaluate the effect of the introduction of the YMHPN. Secondly, we had limited information on specific patient characteristics (e.g., no info on ethnic background of children or the clinical condition of the child). Therefore, we could not investigate differences between specific patient populations. Thirdly, we did not have information on costs for general practice care and medication. As such, we only assessed costs for outpatient mental care. We did not take into account the costs for the implementation of the YMHPN. Future research should address the cost-effectiveness of the YMHPN. Fourthly, participation in the YMHPN-project was not random and it is possible that participating practices differed from those that did not participate. For instance, during our study period the number of practices from deprived areas joining the RPCD and the YMHPN-project increased. However, by adjusting for living in a deprived area we minimalized this confounding effect. Fifthly, not all practices provided follow-up information for all months and in some practices that participated in the YMHPN-project only the period with a YMHPN was covered. We addressed the fourth and fifth limitation by conducting two sensitivity analyses in two subsets of practices (i.e., all practices that participated in the YMHPN-project and all practices with > 3 years follow-up). These sensitivity analyses provided comparable results to our main analysis and thus strengthened our results. Lastly, our study period included the COVID-19 pandemic. Although we corrected for the effect of the COVID-19 pandemic in our models, it cannot be ruled out that some remaining confounding effects did affect our results.

## Conclusion

To our knowledge, the current study is the first large scale research that investigated effects of integrated care, in our case the introduction of a YMHPN in Dutch general practice, on the use of specialized outpatient mental care and associated costs. Our findings indicate small but non-significant decreases of both outcomes across practices. Overall, we did not find significant associations between the presence of YMHPN and outpatient mental care and associated costs within the first four years in which the YMHPN-projects was implemented. We believe more research at both practice and individual patient level is needed to investigate longer-term effects of the introduction of the YMHPN and whether these effects vary for children with different socioeconomic, cultural and ethnic backgrounds in order to gain more insight into the importance of integrated mental care for children.

## Electronic supplementary material

Below is the link to the electronic supplementary material.

Supplementary Material 1

Supplementary Material 2

## Data Availability

No datasets were generated or analysed during the current study.

## Footnotes

1 *In Dutch this position is called ‘praktijkondersteuner jeugd en gezin’ which translates to ‘practice support worker youth and family’ in English. Following previous publications on this position*,* we use the English term ‘youth mental health practice nurse’*.
