# Supplementary material for: Integrating youth mental health practice nurses into general practice: effects on outpatient mental health care utilization among children and adolescents
Source: Eur Child Adolesc Psychiatry. 2024 Dec 12;34(7):2145–54. doi: 10.1007/s00787-024-02619-z (PMC12334438; doi:10.1007/s00787-024-02619-z)
Supplement: Supplementary file 2 — Supplementary Material 2 [file 787_2024_2619_MOESM2_ESM.docx]

**Integrating youth mental health practice nurses into general practice: effects on outpatient mental health care utilization among children and adolescents.**

**Supplementary files**

***eFigure 1 Trends of health care utilization and associated costs per practice over time***

*
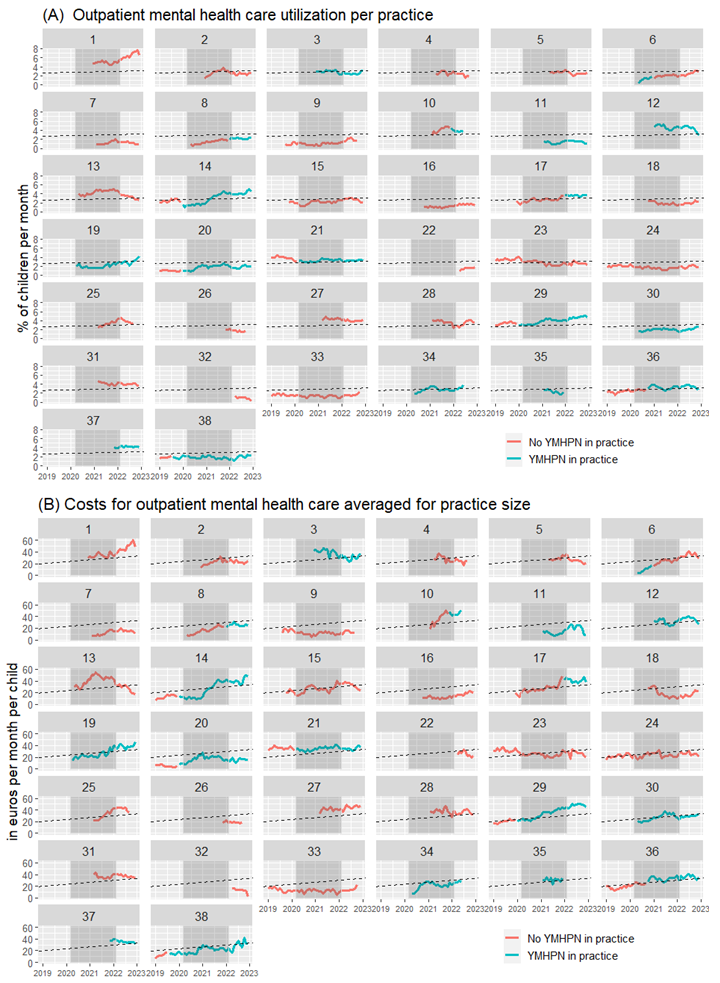
*

** eFigure 1 shows the trends per practice in (a) the monthly percentage of children receiving outpatient mental and (b) average monthly costs for outpatient per registered patient per practice. The dotted-line shows the simple regression for the complete cohort over time. Not all practice provided follow-up data for the complete study period. The color depicts whether a YMHPN was working in the practice. The shaded area shows the Covid-19 pandemic in the Netherlands.*

***eFigure 2 Monthly outpatient mental care utilization per age and social deprivation status over time***


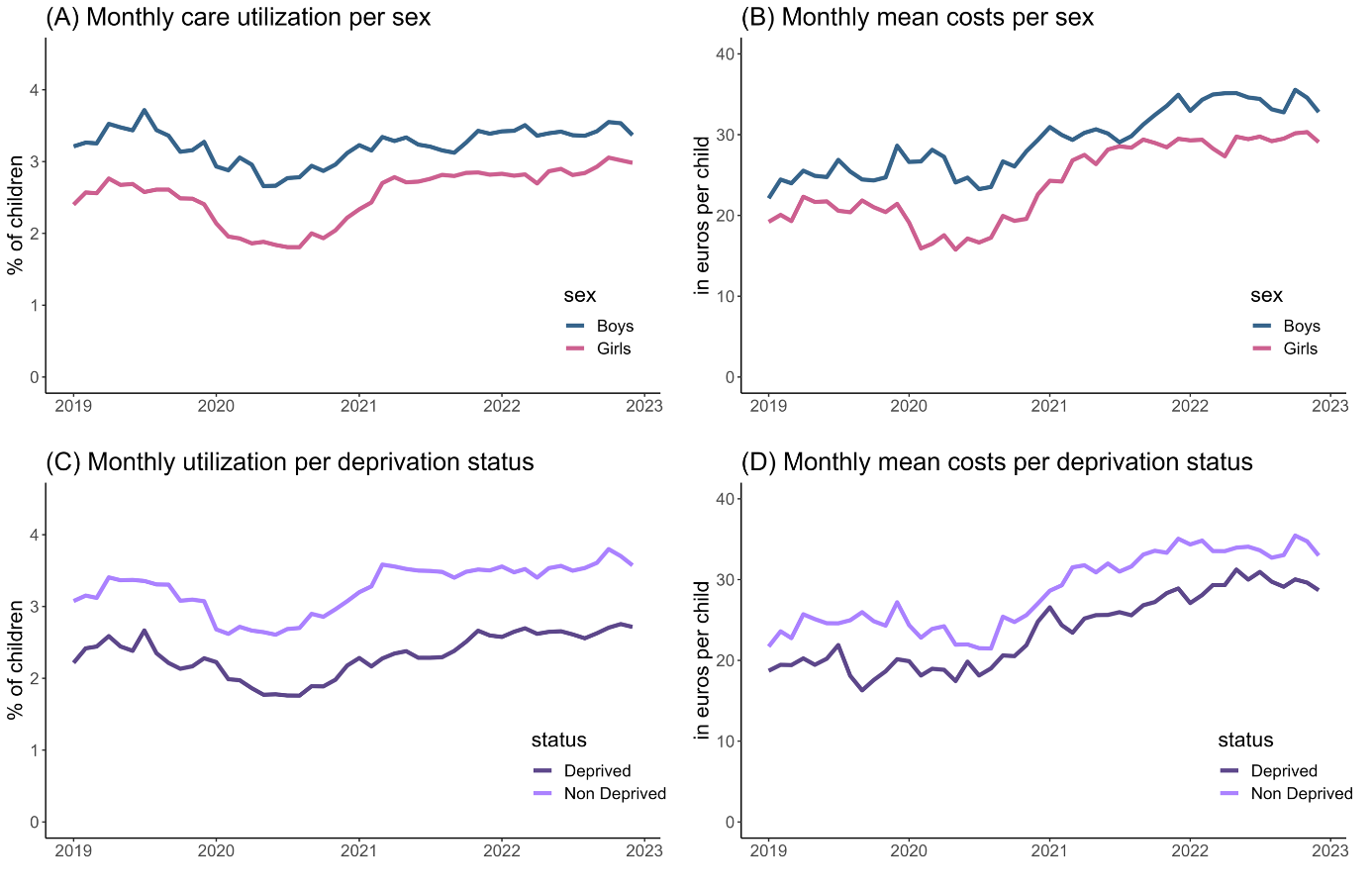


***eTable 1. Sensitivity analyses for Modelled monthly rate of children receiving outpatient mental care per practice***

|  | **Model 1. Modelled monthly rate of children receiving outpatient mental care per practice using complete cohort** | | | **Sensitivity analysis 1: Model 1 in subset of practices that all take part in the YMHPN-project** | | | **Sensitivity analysis 2: Model 1 in subset of practices that provide minimal 36 months of follow-up data** | | |
| --- | --- | --- | --- | --- | --- | --- | --- | --- | --- |
| *Predictors* | *Incidence Rate Ratios* | *CI* | *p* | *Incidence Rate Ratios* | *CI* | *p* | *Incidence Rate Ratios* | *CI* | *p* |
| (Intercept) | 0.02 | 0.02 to 0.03 | <0.001 | 0.02 | 0.02 to 0.03 | <0.001 | 0.02 | 0.02 to 0.03 | <0.001 |
| Monthly trend over time^a^ | 1.00 | 1.00 to 1.01 | 0.024 | 1.01 | 1.00 to 1.02 | <0.001 | 1.01 | 1.00 to 1.01 | 0.012 |
| % Living in social deprived area^b,c^ | 0.99 | 0.99 to 0.99 | <0.001 | 0.99 | 0.99 to 1.00 | <0.001 | 0.99 | 0.98 to 1.00 | 0.011 |
| Mean age in years^c^ | 1.14 | 1.03 to 1.27 | 0.015 | 1.16 | 0.99 to 1.36 | 0.065 | 1.24 | 1.02 to 1.50 | 0.034 |
| % Male ^b,c^ | 1.00 | 0.98 to 1.03 | 0.855 | 1.00 | 0.97 to 1.03 | 0.862 | 1.01 | 0.97 to 1.05 | 0.583 |
| **YMHPN in practice** | 0.99 | 0.92 to 1.06 | 0.695 | 0.94 | 0.87 to 1.02 | 0.123 | 0.98 | 0.90 to 1.07 | 0.641 |
| First Covid-19 wave ^d^ | 0.88 | 0.84 to 0.92 | <0.001 | 0.89 | 0.84 to 0.95 | <0.001 | 0.89 | 0.84 to 0.94 | <0.001 |

The estimates are adjusted for practice size by using the log of the number of children per practice as offset variable. ^a^Modelled linear trend over time (range 0-47) every month increase over time is associated with increase in expected costs. ^b^In percentage per practice, the shown estimate is the increase in costs per percentage increase of the variable (eg 40 to 41% percentage of children living in deprived area) ^c^These variables are centered on the grand mean average (eg, average percentage of children living in social deprived area). ^d^Dummy variable to correct for the period of march 2020 to December 2020 during which a strong decrease in mental health care utilization was observed.

***eTable 2. Sensitivity analyses for modelled average monthly costs for outpatient mental health care per practice***

|  | **Model 2. Modelled monthly costs for outpatient mental care per practice using complete cohort** | | | **Sensitivity analysis 3: Model 2 in subset of practices that all take part in the YMHPN project** | | | **Sensitivity analysis 4: Model 2 in subset of practices that provide minimal 36 months of follow-up data** | | |
| --- | --- | --- | --- | --- | --- | --- | --- | --- | --- |
| *Predictors* | *Estimates* | *CI* | *p* | *Estimates* | *CI* | *p* | *Estimates* | *CI* | *p* |
| (Intercept) | 16790.19 | 12946.46 to 20633.91 | <0.001 | 19395.94 | 12827.74 to 25964.14 | <0.001 | 15736.84 | 10417.34 to 21056.34 | <0.001 |
| Monthly trend over time^a^ | 125.03 | 8.22 to 241.83 | 0.036 | 239.49 | 42.42 to 436.57 | 0.017 | 240.79 | 13.37 to 468.20 | 0.038 |
| % Living in social deprived area^b,c^ | -111.78 | -165.91 to -57.64 | <0.001 | -78.21 | -151.82 to -4.61 | 0.037 | -52.91 | -132.80 to 26.99 | 0.194 |
| Mean age in years^c^ | 3412.38 | 1755.19 to 5069.58 | <0.001 | 4264.55 | 1536.53 to 6992.56 | 0.002 | 3431.90 | 1286.88 to 5576.93 | 0.002 |
| % Male ^b,c^ | -42.40 | -354.51 to 269.72 | 0.790 | -232.43 | -698.54 to 233.67 | 0.328 | 296.13 | -135.73 to 727.99 | 0.179 |
| **YMHPN in practice** | **-395.80** | **-1431.27 to 639.67** | **0.454** | **-575.86** | **-1787.18 to 635.46** | **0.351** | **-960.13** | **-2205.74 to 285.48** | **0.131** |
| Number of registered children in practice^c^ | 34.55 | 30.58 to 38.51 | <0.001 | 34.48 | 30.21 to 38.74 | <0.001 | 35.25 | 31.47 to 39.03 | <0.001 |
| First Covid-19 wave ^d^ | -2333.39 | -2931.19 to  -1735.60 | <0.001 | -2753.31 | -3630.85 to -1875.77 | <0.001 | -1803.15 | -2511.27 to -1095.04 | <0.001 |

^a^Modelled linear trend over time (range 0-47) every month increase over time is associated with increase in expected costs. ^b^In percentage per practice, the shown estimate is the increase in costs per percentage increase of the variable (eg 40 to 41% percentage of children living in deprived area) ^c^These variables are centered on the grand mean average (eg, average number of children registered per practice). ^d^Dummy variable to correct for the period of march 2020 to December 2020 during which a strong decrease in mental health care utilization was observed.
